# The Prevalence of Incidentally Detected Urolithiasis in Subjects Undergoing Computerized Tomography

**DOI:** 10.7759/cureus.10374

**Published:** 2020-09-11

**Authors:** Sajeel Saeed, Ansar Ullah, Jawad Ahmad, Sidra Hamid

**Affiliations:** 1 Surgery, Rawalpindi Medical University, Rawalpindi, PAK; 2 Physiology, Rawalpindi Medical University, Rawalpindi, PAK

**Keywords:** epidemiology, urolithiasis, calculi, computerized tomography, urinary tract stones

## Abstract

Background and objective

Urolithiasis is defined as the presence of calculi in the urinary tract. Multiple studies have shown that urinary tract stones are one of the most common incidental findings in medical imaging. These stones are potentially dangerous and can cause severe impairment to renal function if they remain undiagnosed for a long time. The objective of this study was to determine the prevalence of incidentally detected urolithiasis in patients undergoing abdominopelvic CT scans.

Materials and methods

A retrospective cross-sectional study was conducted, which involved 721 patients selected by consecutive non-randomized sampling. The study population included patients who underwent an abdominopelvic CT scan in the radiology department of a tertiary care hospital in Pakistan. Patients aged below 10 years and those above 90 years were excluded from the study. Patients undergoing kidney, ureter, and bladder (KUB) scan for urolithiasis-associated symptoms and those with already known urolithiasis were also excluded. The data were recorded in a predesigned pro forma and analyzed with SPSS Statistics version 20.00 (IBM, Armonk, NY).

Results

A total of 721 patients underwent an abdominopelvic CT scan during the six months from July to December in the radiology department of the hospital. Out of these, 336 (46.6%) were males, and 385 (53.4%) were females. Incidental stones were found in 20 of these patients. Among these 20 stone formers, 11 were males, and nine were females. Out of them, six had stones in the right kidney, eight in the left kidney, and four patients had bilateral stones. The remaining two patients had stones in their ureters. In most cases, stones were found in lower poles as compared to the mid pole and upper pole of the kidneys.

Conclusion

The prevalence of incidentally detected urolithiasis was found to be 2.8% in this study. Its frequency was much higher in males (3.27%) compared to females (2.33%). Most of the stones were found in the kidneys whereas no stone was detected in the urinary bladder.

## Introduction

Urolithiasis is a polygenic and multifactorial disorder with a prevalence of about 2-20% worldwide [1,2], and it is becoming a serious problem as being the third most frequent urinary tract disease, surpassing urinary tract infections (UTI) and prostatic pathology [1]. The prevalence of urolithiasis has been on the rise in recent years. In Asia, its prevalence is around 1-5%, whereas, in North America and Europe, its prevalence is about 7-13% and 5-9%, respectively [3]. A study in Pakistan has also revealed a 3% prevalence and a 5% probability of silent stones in the general population [4].

There is a "stone-forming belt" stretching across Asia with a very high prevalence of urolithiasis ranging from 1-19%, the highest being in countries like Japan and South Korea, where the prevalence is 8-19%. The prevalence of urolithiasis in countries lying in this stone belt has increased over the past few decades. On the one hand, this rise in prevalence is attributed to the change in climate and dietary habits, while, on the other hand, the increase in the usage and sensitivity of imaging techniques is also responsible for this apparent increase in the reported prevalence of urolithiasis [5].

Signs and symptoms of urinary tract stones include severe pain that radiates from the flank region to the groin. This pain, known as renal or ureteric colic, is one of the most severe pain sensations known. The condition is also marked by urinary urgency, restlessness, hematuria, nausea, vomiting, and sweating.

Stones can remain clinically silent for years. However, when they grow beyond a size that allows for spontaneous clearance through the urinary tract, they may cause severe damage to kidney structure and impairment to renal function. About 77% of these asymptomatic stones show disease progression [6]; if they remain undiagnosed, these stones can cause permanent damage to kidney function. Most of these stones eventually require surgical intervention [6,7]. Ultrasonography is an easy-to-use and cost-effective diagnostic method used for detecting urolithiasis, but studies have shown that it has less sensitivity (50%) and often results in size overestimation [8]. In light of this, CT has become the most preferred method, and it has proven to be the gold standard for detecting urinary tract stones [9].

The objective of this study was to determine the prevalence of incidental urinary tract stones in patients undergoing CT scans. Further, we believe the study will help in creating awareness among medical practitioners regarding the previously underestimated prevalence of asymptomatic urinary tract calculi.

## Materials and methods

A descriptive cross-sectional study was conducted in the radiology department of a tertiary care hospital. This study was conducted from July 2019 to December 2019 using consecutive non-randomized sampling. Patients undergoing abdominopelvic CT scans in the radiology department were included in the study irrespective of gender. All patients, regardless of whether they had symptoms of UTI, were included in the study. All patients aged above 90 years and those below 10 years were excluded from the study. Furthermore, all patients undergoing kidney, ureter, and bladder (KUB) scans for urolithiasis-associated symptoms and those with already known urolithiasis were also excluded. Moreover, subjects undergoing dialysis were also excluded.

A total of 721 patients underwent CT scans in the radiology department of our hospital during the study period. Demographic details were taken either from the patients or from the hospital records of CT scans. The age of the patients along with sex, clinical indication, size and diameter of the stone, number of stones, location concerning the ureter, urinary bladder, and upper, middle, and lower poles of both kidneys were recorded. On the basis of patient age, the study population was classified into four categories: group 1 (10-30 years of age), group 2 (31-50 years of age), group 3 (51-70 years of age), and group 4 (71-90 years of age).

All data were recorded in a predesigned pro forma. Data were entered and analyzed via the SPSS Statistics version 20.00 (IBM, Armonk, NY). The mean values and standard deviation were recorded for the quantitative variables such as age, as given in Table 1, which included stone bearers as well as non-stone bearers. The frequency of stones, along with their location and laterality, was also determined. The Kolmogorov and Shapiro-Wilk tests were used to check the normality of the quantitative variables such as age.

Before the commencement of the study, the Institutional Research Forum of Rawalpindi Medical University issued ethical approval (RSRS-2019-S-011). Furthermore, ethical approval was also taken from the surgical department of the tertiary care hospital for the collection of data for this study.

## Results

A total of 721 patients underwent CT scans of the abdomen and pelvis during the six months from July to December 2019. Of these patients, 336 (46.6%) were males, and 385 (53.4%) were females. Table 1 shows the demographic details of patients with or without stones.

**Table 1:** Demographic details of the patients *Figures in parentheses represent standard deviation

Variables	Values
Total number of patients	721
Total number of patients having stones	20
Overall mean age of patients, years	44.89 (± 17.1)*
Mean age of stone-bearer patients, years	51.8 (± 14.0)*
Mean age of patients without stones, years	44.69 (± 17.2)*

As shown in Table 1, the overall mean age of the patients was 44.89 ± 17.131 years. The mean age was 45.18 ± 17.575 years for males and 44.63 ± 16.752 years for females. About 70% of the population undergoing CT scans was within the age range of 31-70 years, whereas the number of patients above 70 years was negligible.

As given in Table 2, the most common clinical indication for CT scan was abdominal pain, which was present in about 33% of the total sample size. Hepatobiliary disease, gastrointestinal disease, cancer, and ascites were the clinical indications in 17.9%, 14.6%, 13.3%, and 5.4% of the study population respectively, whereas fever, accident, hematological causes, postsurgical, respiratory diseases, genital disorders, musculoskeletal disorders, and generalized edema were the least common conditions for which CT scans were advised.

**Table 2:** Clinical indications of patients undergoing abdominopelvic CT CT: computed tomography

Clinical indications	Presence of stones		Total number of people
	Present	Absent	
Hepatobiliary disease	7	98	105
Fever	0	25	25
Trauma	0	13	13
Gastrointestinal disease	5	124	129
Genitourinary disorders	0	9	9
Cancer	1	95	96
Musculoskeletal problems	2	9	11
Respiratory disease	2	11	13
Abdominal pain	1	237	238
Hematological disease	1	15	16
Ascites	0	39	39
Generalized edema	1	8	9
Postsurgical problems	0	16	16
Causes not known	0	2	2

Out of the 721 patients, stones were detected incidentally in 20 patients. The prevalence of stones was highest in the age group of 31-70 years as 18 out of 20 stone forms were found within this age group. As given in Table 1, the mean age of the stone bearers was 51.80 ± 14.00 years, and for non-stone bearers, the mean age was 44.69 ± 17.9 years. One patient above 70 years of age and one below 30 years old had a stone. Out of the total 20 stone formers, 11 were males and nine were females. The mean age of male and female stone formers was 52.8 ± 15.8 years (range: 20-80 years) and 50.7 ± 12.3 years (range: 35-70 years), respectively.

As shown in Table 3, calculi were found on the right side in seven patients, on the left side in nine, and bilaterally in four patients. Out of them, six patients had stones in the right kidney, eight had in the left kidney, and four patients had bilateral stones. The remaining two patients had stones in their ureters.

**Table 3:** Laterality-wise location of stones in the urinary tract

Laterality-wise location	Location of stones			Total
	Kidney	Ureter	Bladder	
Right side	6	1	0	7
Left side	8	1	0	9
Both sides	4	0	0	4
Total	18	2	0	20

No stone was found in the patients who were advised a CT scan due to fever, ascites, trauma, genitourinary disorders, and postsurgical problems. In contrast, the patients undergoing CT scan due to gastrointestinal, hepatobiliary, respiratory, hematological, and musculoskeletal problems had stones, with the highest prevalence among people with deranged musculoskeletal, respiratory, hepatobiliary, and gastrointestinal systems, accounting for a prevalence of 22%, 18%, 7%, and 4%, respectively.

Out of the 20 patients with urolithiasis, 11 had a single stone, one had two stones, two patients had three stones, while one patient had four stones in the urinary tract. Out of these 23 stones, six were found in the lower pole of the right kidney, five in the lower calyx of the left kidney, four in the inter-polar region of the left kidney, three in the inter-polar region of the right kidney, and one at the upper pole of the right and left kidneys each, whereas two stones were found in the renal pelvis. The remaining five patients had multiple stones with exact numbers not known. Excluding these patients with multiple stones, the rest had 1.53 stones on average.

Only five patients had a stone with a size above 1 cm. The most massive stone detected was 1.9 cm in size, which was found in the right kidney. Ureteric stones had an average size of 7.2 mm (AP).

## Discussion

Urolithiasis is a serious healthcare burden globally. It is characterized by the presence of calculi anywhere in the kidney, including the upper, middle, or lower calyx of the kidney, renal pelvis, the proximal, middle, and distal part of the ureter, and in the urinary bladder. Most commonly, complete blood picture, as well as renal function tests, are performed before the actual diagnostic procedures. To confirm a stone, several diagnostic procedures, including ultrasound or CT scan, are performed. Ultrasound is usually done in emergencies, as it is advantageous in certain conditions. It is less expensive and emits no ionizing radiation. It also has a reasonable sensitivity for identifying indirect stones, especially non-obstructing stones of hydro-nephrosis. Nevertheless, it is not the gold standard for detecting urinary tract stones. It has less sensitivity and specificity than CT scans.

CT scan is currently the gold standard for detecting urolithiasis [9]. It has a specificity of 94-99% and a sensitivity of 95-98% for detecting calculi [10]. Additionally, it can easily differentiate between appendicitis and urolithiasis as appendicitis can mimic stone disease [11]. MRI can also be used for detecting urolithiasis as it has zero radiation exposure problems, but it is very expensive, i.e., it costs approximately three times more than a CT scan. Moreover, although its specificity is comparable to that of a CT scan (98%), its sensitivity is 82%, which is much less than that of the CT scan [12]. Hence, due to these reasons, the CT scan is the most appropriate method for the diagnosis of urolithiasis.

The worldwide prevalence of urolithiasis is about 2-20% [1,2]. It is the most common urological problem in areas with a warm and dry climate [13]. The temperature in Pakistan is reasonably high due to which Pakistan is included in the region called "the stone-forming belt" by some authors [14]. In Pakistan, the prevalence of urolithiasis is about 1-5%. In our study, the prevalence of asymptomatic stones was found to be about 2.8%, which is similar to the study results of researches conducted previously [4]. Usually, stones remain silent initially, but as time passes, it starts inducing symptoms to the stone bearer. Symptoms usually include flank pain (renal colic), urinary urgency, restlessness, vomiting, hematuria, and nausea. Studies have shown that about one in five patients shows symptoms of urinary tract stones during a 10-year life span [15]. Risk factors for increased urinary tract stone incidence include a family history of nephrolithiasis, warm climate, dehydration or low water intake, obesity or high BMI, increased dietary intake of proteins, and hyperparathyroidism.

The lifetime chance of getting a stone is about twice in males as compared to females; males have a 10% chance of having a stone, whereas it is about 5-10% in females [16]. In our study, the prevalence of incidental urolithiasis was higher in males compared to females, as the former had a frequency of about 3.27%, whereas the latter had a frequency of 2.33%. This was consistent with a study conducted by Nassir [17], where the prevalence was 6.6% among males and 5.8% among females. Another study by Buchholz et al. found all stone formers to be males [4]. A study by Menon et al. has reported that men are three times more vulnerable to urolithiasis than women [18]. This may be because men are more exposed to hot climates and more prone to developing dehydration. Furthermore, the dietary habits of men, such as the propensity for salt and protein intake, may also contribute to an increased frequency of stones among them. Moreover, the increased prevalence of stones among men becomes even more evident as the total number of women (385) included in the study was higher as compared to men (336).

The age group most affected by stones was that of 30-70 years. Males and females were both affected by the same frequency in this age group. The mean age of stone bearers was 51.80 ± 14.00 years, which is comparable to another study, where the mean age of stone formers was 48.4 ± 17.6 years [4]. As the study population was divided into four groups based on age (10-30, 30-50, 50-70, and 70-90 years), all age groups had stone prevalence among them; however, the most affected age group among these was that of 50-70 years, where the prevalence was about 4.2%. The National Health and Nutrition Examination Survey (NHANES II) has also shown an increased frequency of stones among the age group of 60-70 years in both men and women [19]. The reason for older adults being prone to urolithiasis could be due to medication supplementations, including vitamins, calcium tablets, hormonal factors, etc. Moreover, obesity and alcohol abuse are also linked with increased calculi in the elderly age group.

In our study, nine (55%) patients had stones on the left side, and seven (45%) had on the right side. Four patients had bilateral stones. The study conducted by Ketata et al. found 44% of the stones on the right side, and 56% of the stones on the left side [20]. Buchholz et al. also reported silent stones to be located predominantly on the left side [4]. The number of stones, as well as laterality, found in our study is given in Table 3.

Out of the total of 20 stones, 18 were found in the kidneys, and two were ureteric. This shows that ureteric stones can be clinically silent. This is supported by a study conducted by Wimpissinger et al. [21], who also supported the idea that ureteric stones can be asymptomatic. Patients having a stone in the ureter presented with the problems of hepatobiliary disease [hepatitis C virus (HCV)-related decompensated liver cirrhosis (DLC)] and gastrointestinal problems (abdominal distension). Ureteric stones were causing mild dilation of the pelvicalyceal system on their respective sides in both patients. These ureteric stones had an average size of 7.2 mm and an average CT density of 1094 HU.

Eleven patients had single stones while the rest showed more than one stone on the abdominopelvic CT scan. This indicates that 55% of the stone bearers had a single stone, whereas 45% of the patients had multiple stones. No pattern of distribution could be developed for the distribution of single and multiple stones. This is supported by other studies conducted previously [4]. Most of the patients in our study had stones in the lower renal calyx, followed by the inter-polar region, upper calyx, and renal pelvis. The study conducted by Cass et al. had also shown a high incidence rate of about 25-35% of lower-pole calculi [22].

Stone disease is beginning to exert a significant economic burden on Pakistan. Urolithiasis is the sixth most common condition requiring surgery in Pakistan [23]; 18% of outpatients presenting in the urological unit report having urinary tract stones [24], and the treatment of urolithiasis accounts for 45% of the workload in a urological unit (Abstract: Sheikh M. Quest For High Technology. Asian Congress of Urology, Bangkok, Asian Urological Association, 1994). In South Asia, 2,619 potential life years are lost due to renal disease, including urolithiasis, whereas the whole world loses 11,415 life years [25]. With improved facilities, this incidence rate can be lowered. Even though the prevalence of these "incidentally found urinary tract stones" was found to be low in our study, we recommend that further studies be conducted on this topic so that a clearer and more comprehensive picture can be obtained.

## Conclusions

The prevalence of incidentally detected urolithiasis was found to be 2.8% in our study. Its frequency was much higher in males (3.27%) compared to females (2.33%). Of note, 90% of these stones were found in the kidneys, whereas the remaining stones were found in the ureter. No stone was found in the urinary bladder. Timely detection of asymptomatic urinary tract calculi through conventional screening could potentially save a large proportion of our population from experiencing the excruciating pain of renal colic later on in life.
